# Personality Traits, Psychiatric Symptoms, and Glycemic Control in Patients with Type 2 Diabetes Mellitus: A Cross-Sectional Study

**DOI:** 10.3390/healthcare14101274

**Published:** 2026-05-08

**Authors:** Necip Nas, Fatih Saglam

**Affiliations:** 1 Department of Internal Medicine, Medical Faculty, Siirt University, 56100 Siirt, Turkey; 2 Department of Psychiatry, Siirt Training and Research Hospital, 56100 Siirt, Turkey; aga.asa@hotmail.com

**Keywords:** diabetes mellitus, personality belief questionnaire, personality traits, psychiatric symptoms, psychosocial factors, symptom checklist-90

## Abstract

**Background:** Diabetes mellitus (DM) management is influenced by both physiological and psychosocial factors. Personality traits and psychiatric symptoms may affect glycemic control, but their exact relationship remains unclear. This study aimed to evaluate the association between glycemic control, personality traits, and psychological symptoms such as depression, anxiety, and stress in patients with DM. **Methods:** A total of 174 adult patients with Type 2 DM (T2DM) were included. Glycemic control was assessed via HbA1c values over one and five years. Personality traits were measured using the Personality Belief Questionnaire-Short Form (PBQ-SF), and psychiatric symptoms were evaluated with the Symptom Checklist-90-Revised (SCL-90-R). Patients were grouped based on HbA1c levels: good (<7%), moderate (7–9%), and poor (>9%) control. **Results:** Significant differences were found in several personality traits, including obsessive-compulsive, histrionic, paranoid, passive-aggressive, antisocial, and schizoid traits, between groups, with higher scores in patients with moderate glycemic control compared to those with poor control. No significant relationship was observed between psychiatric symptoms and glycemic control. The proportion of patients meeting the predefined HbA1c follow-up frequency threshold was lowest in the poor control group. **Conclusions:** General psychiatric symptoms did not show significant differences across glycemic control groups. Several maladaptive personality traits differed between groups in univariate analyses; however, after adjustment for potential confounders, only passive-aggressive personality traits remained independently associated with HbA1c levels. These findings indicate a possible association between specific personality characteristics and glycemic control, although no direct clinical utility can be inferred from the present data. Further longitudinal studies are required to clarify these relationships.

## 1. Introduction

Diabetes Mellitus (DM) is a serious metabolic disease with a chronic course characterized by elevated plasma glucose levels (hyperglycemia) caused by a deficiency in the release of insulin produced in the body or insulin resistance in tissues [1]. The global increase in diabetes cases is influenced by factors such as genetics, aging, lifestyle, and rising body mass index (BMI) in the population. As diabetes becomes increasingly common, understanding the multifactorial nature of the disease, including both physiological and psychosocial components has become essential for effective prevention and intervention strategies [2].

Personality refers to relatively stable patterns of perceiving, thinking, and behaving that shape an individual’s interaction with the environment [3]. Personality traits represent dimensional characteristics that vary across individuals, whereas personality disorders are categorical diagnoses defined by persistent and maladaptive patterns that significantly impair functioning [4]. These constructs are related but not interchangeable and should be considered separately in research contexts.

Personality disorders, as defined by the Diagnostic and Statistical Manual of Mental Disorders, Fifth Edition (DSM-5), are grouped into three clusters: A (odd/eccentric), B (dramatic/emotional), and C (anxious/fearful). Although these diagnostic categories provide a clinical framework, this study does not aim to diagnose personality disorders. Instead, it focuses on maladaptive personality-related beliefs and traits that may influence health behaviors. Cluster A includes Paranoid, Schizoid, and Schizotypal types, Cluster B includes Antisocial, Borderline, Histrionic, and Narcissistic types, and Cluster C includes Avoidant, Dependent, and Obsessive-Compulsive types [5].

Type 2 DM (T2DM) is a complex chronic condition influenced not only by physiological but also psychological and behavioral factors [6,7]. In chronic disease management, personality characteristics, illness perception, and engagement with treatment play important roles [8]. In this context, the Personality Belief Questionnaire-Short Form (PBQ-SF) is used to assess dysfunctional beliefs associated with personality traits rather than to establish clinical diagnoses of personality disorders.

Therefore, the aim of this study was to evaluate the association between glycemic control, as measured by HbA1c levels, and personality-related belief patterns and psychiatric symptoms in individuals with T2DM. We hypothesized that maladaptive personality-related beliefs would be associated with differences in glycemic control, whereas general psychiatric symptom severity would show a weaker or non-significant association.

## 2. Materials and Method

### 2.1. Ethical Approval

This study was approved by the Ethics Committee of Siirt University (Decision No: 72518; Date: 5 April 2023) and conducted in accordance with the Declaration of Helsinki. Given the retrospective nature of the study and the use of anonymized data, the requirement for written informed consent was waived by the Ethics Committee.

### 2.2. Study Design

This study was designed as a retrospective cross-sectional observational study. Retrospective clinical data, including HbA1c measurements over 1-year and 5-year periods, were obtained from electronic medical records, while personality-related beliefs and psychiatric symptoms were assessed cross-sectionally through face-to-face interviews at a single time point.

### 2.3. Study Populations

Patients were recruited consecutively among those attending the diabetes outpatient clinic who met the inclusion criteria. This retrospective study was conducted between January 2013 and January 2023, and HbA1c values were analyzed over predefined reference periods of 1 year (short-term glycemic control) and 5 years (long-term glycemic control). This study included a total of 174 adult patients (aged 18 years and older) diagnosed with T2DM who were being followed at the diabetes outpatient clinic of Siirt Training and Research Hospital. HbA1c values over the past one and five years were retrospectively reviewed. One-year HbA1c data was available for all 174 patients, while five-year data was available for 165 of them. Face-to-face interviews were conducted to administer the questionnaires. Treatment regimens included oral hypoglycemic agents, insulin therapy, or a combination of both. Demographic characteristics, including age, sex, smoking status, alcohol consumption, living alone, and exercise habits, as well as medical histories, were obtained through structured questionnaires and a review of medical records.

T2DM was defined as per the American Diabetes Association (ADA) criteria. The average HbA1c levels were calculated to assess long-term and short-term glycemic control, which is a widely accepted marker for diabetes management and treatment outcomes [9]. Glycemic control status was evaluated using HbA1c levels obtained from short-term clinical follow-up. Patients were classified as having mild hyperglycemia if HbA1c was below 7%, moderate if between 7% and 9%, and severe if above 9%. Glycemic control (GC) was considered good when HbA1c was less than 7.0%, and poor when it exceeded 7.0% [10].

In addition, data on psychiatric treatment history, type of antidiabetic therapy, follow-up engagement, and sociodemographic characteristics (age and sex) were collected. Due to limitations inherent to the retrospective medical records, antidiabetic treatment could not be consistently stratified according to specific drug classes (e.g., DPP-4 inhibitors, SGLT2 inhibitors, GLP-1 receptor agonists) or detailed insulin regimens (basal vs. basal-bolus) for all participants; therefore, therapies were categorized into broader groups (oral agents, insulin therapy, or combination therapy). Treatment category was included in multivariable analyses to partially account for potential confounding related to therapeutic escalation.

Psychiatric symptoms were assessed using the Symptom Checklist-90-Revised (SCL-90-R), and personality-related belief patterns were evaluated using the Personality Belief Questionnaire-Short Form (PBQ-SF). The PBQ-SF is designed to assess dysfunctional beliefs associated with personality dimensions and does not provide a clinical diagnosis of personality disorders; therefore, findings should be interpreted within the framework of personality-related belief patterns rather than formal personality traits or disorders.

Follow-up engagement was operationalized using HbA1c testing frequency. Patients with at least two HbA1c measurements per year were classified as meeting the predefined follow-up frequency threshold, whereas those with fewer than two annual measurements were classified as below this threshold. This variable was used as an indirect indicator of healthcare utilization and should be interpreted with caution, as it may reflect multiple factors beyond patient adherence, including clinical practice patterns and disease severity.

Eligible patients were initially identified retrospectively through the hospital electronic medical records based on a confirmed diagnosis of T2DM and availability of HbA1c data. Subsequently, patients were contacted using the information recorded in the hospital system and were invited to participate in face-to-face interviews during their routine outpatient clinic visits.

### 2.4. The Inclusion Criteria

Adults aged 18 years or older with a confirmed diagnosis of T2DM, who had available HbA1c measurements in the medical records and agreed to participate in face-to-face interviews by completing the study questionnaires were included.

### 2.5. The Exclusion Criteria

Patients with conditions that could interfere with reliable data collection or questionnaire completion were excluded. These included individuals with moderate to severe dementia, blindness, or severe psychiatric disorders (e.g., bipolar disorder and schizophrenia). Patients with incomplete clinical data or missing HbA1c records were also excluded.

### 2.6. Measurement Instruments

The PBQ is a 126-item self-report inventory developed by Aaron Beck and Judith Beck to assess dysfunctional beliefs associated with personality disorders [11]. The questionnaire aims to evaluate nine personality traits: avoidant, dependent, passive-aggressive, obsessive-compulsive, antisocial, narcissistic, histrionic, schizoid, and paranoid. Respondents rate each item on a scale from 0 to 4 (0 = do not believe at all, 4 = believe completely). A shortened version consisting of 65 items was later developed. The Turkish version of the original PBQ was evaluated for its validity and reliability by Turkcapar in 2007 [12].

The SCL-90-R was developed by Derogatis and consists of 90 items [13]. The scale assesses nine symptom dimensions: somatization, obsessive-compulsive, interpersonal sensitivity, depression, anxiety, hostility, phobic anxiety, paranoid ideation, and psychoticism. In addition to these subscales, a Global Severity Index (GSI) can also be calculated to reflect overall psychological distress. Each item is rated on a 5-point Likert scale ranging from 0 (not at all) to 4 (extremely).

HbA1c determination was based on HPLC (Variant Turbo II, Bio-Rad Laboratories, Inc., Hercules, CA, USA).

### 2.7. Statistical Analysis

Data analyses were performed using SPSS version 20.0 (Statistical Package for the Social Sciences, Chicago, IL, USA). The sample size of our study was determined using G*Power software (Version 3.1.9.7). Power was set at 95%, and alpha and effect size were kept as 0.01 and 0.15, respectively. According to this analysis, the smallest sample size was determined as 157. Dependent Variable: The primary outcome was glycemic control status, which was operationalized based on HbA1c levels (categorized as <7% for good control vs. ≥7% for suboptimal control). Independent Variables: The primary independent variables were personality traits as measured by the PBQ-SF subscales. Covariates (Adjusting Variables): To minimize potential confounding, the multivariable model included age, sex, duration of diabetes, BMI, treatment modality (e.g., oral agents, insulin, or mixed therapy), and comorbidities as covariates. Categorical variables were presented as frequencies (*n*) and percentages (%), while continuous variables were expressed as mean ± standard deviation (SD). For comparisons between two groups, Student’s *t*-test was used for normally distributed variables, and the Mann–Whitney U test was applied for non-normally distributed variables. One-way ANOVA followed by Tukey’s post hoc test was used for comparisons among more than two groups. For categorical variables, the Chi-square test or Fisher’s exact test was used, depending on the sample size. Univariate and Multivariable Binary Logistic Regression analyses were performed to determine the relationship between HbA1c levels and personality traits. The models were adjusted for age, sex, diabetes duration, BMI, treatment modality, renal function (eGFR), and presence of diabetes-related complications to minimize potential confounding by disease severity and treatment intensity. A *p*-value of <0.05 was considered statistically significant.

## 3. Results

Of the 174 patients included in the study, 123 were female and 51 were male. Based on their average HbA1c levels, patients were categorized into three groups: Group 1 included those with HbA1c levels below 7, Group 2 included those with HbA1c levels between 7 and 9, and Group 3 included those with HbA1c levels of 9 or higher. No statistically significant differences were found among the three groups in terms of demographic variables such as age and gender (*p* > 0.05).

In the statistical analysis based on the average HbA1c levels over the past year, no significant differences were found among the 174 patients in terms of SCL-90-R symptom dimensions (*p* > 0.05). However, when the three groups were compared using the Personality Belief Questionnaire, significant differences were observed in the Obsessive-Compulsive Personality (*p* = 0.041), Histrionic Personality (*p* = 0.022), and Paranoid Personality (*p* = 0.047) subscales, as determined by one-way ANOVA. Tukey’s post hoc test was used to determine the direction of these differences. In all three personality subscales, Group 2’s scores were significantly higher than those of Group 3 (Obsessive-Compulsive Personality: *p* = 0.033; Histrionic Personality: *p* = 0.036; Paranoid Personality: *p* = 0.035). Comparison of HbA1c follow-up patterns showed that Group 2 had the highest rate of patients meeting follow-up criteria (12.6%), while Group 3 had the highest rate of patients not meeting the criteria (36.2%). The most used treatment method among all patients was mixed therapy (oral antidiabetics + insulin), applied in 51.7% of cases (*p* < 0.001). No significant differences were observed between the groups in terms of psychiatric treatment or comorbid medical conditions. The main characteristics and intergroup comparisons of the variables are summarized in Table 1.

In the statistical analysis based on the 5-year average HbA1c values, no significant differences were found among the 165 patients in terms of SCL-90-R symptom dimensions (*p* > 0.05). However, comparison of the Personality Belief Questionnaire scores across the three groups revealed significant differences in the Passive-Aggressive Personality (*p* = 0.008), Obsessive-Compulsive Personality (*p* = 0.012), Antisocial Personality (*p* = 0.034), Schizoid Personality (*p* = 0.042), and Paranoid Personality (*p* = 0.003) subscales, as determined by one-way ANOVA. Tukey’s post hoc test was applied to determine the direction of the differences. In all five personality subscales, Group 2 had significantly higher scores compared to Group 3 (Passive-Aggressive: *p* = 0.006; Obsessive-Compulsive: *p* = 0.009; Antisocial: *p* = 0.029; Schizoid: *p* = 0.036; Paranoid: *p* = 0.002). Comparison of HbA1c follow-up frequency showed that Group 2 had the highest rate of patients meeting follow-up criteria (13.9%), while Group 3 had the highest rate of patients below the follow-up threshold (37.6%). The most used treatment method was mixed therapy (oral antidiabetics + insulin), applied in 52.1% of the patients (*p* < 0.001). No statistically significant differences were found between the groups in terms of psychiatric treatment or comorbid medical conditions. The key features and intergroup comparisons of the variables are summarized in Table 2.

Additionally, when examining the relationship between HbA1c follow-up compliance and psychiatric treatment, no statistically significant difference was found in both the 1-year and 5-year patient groups.

To determine the personality traits associated with HbA1c levels, a regression analysis was performed after adjusting for age, gender, duration of diabetes, treatment regimen, and comorbidities. In the univariate regression analysis, passive-aggressive personality (OR, 1.12 per point increment; 95% CI, 1.04–1.17), obsessive-compulsive personality (OR, 1.36 per point increment; 95% CI, 1.15–3.27), antisocial personality (OR, 1.74 per point increment; 95% CI, 1.05–3.18), schizoid personality (OR, 2.12 per point increment; 95% CI, 1.18–3.72), and paranoid personality (OR, 2.47 per point increment; 95% CI, 1.64–3.87) were identified as personality traits associated with HbA1c levels. In the multivariate regression analysis, Passive-Aggressive Personality (OR, 1.12 per point increment; 95% CI, 1.01–1.18) was determined to be independently associated with the HbA1c level (Table 3).

## 4. Discussion

The present study demonstrates an association between specific maladaptive personality-related belief patterns and glycemic control in patients with T2DM. Patients with moderate glycemic control (HbA1c 7–9%) exhibited higher scores in several dimensions, including obsessive-compulsive, histrionic, paranoid, passive-aggressive, antisocial, and schizoid patterns, compared with those with poor glycemic control (HbA1c ≥9%). This counterintuitive finding may suggest that individuals with moderate control exhibit more structured yet rigid or stress-sensitive approaches to disease management. However, this interpretation remains speculative, as no direct measures of treatment adherence, self-management behaviors, or engagement with care were assessed in the present study. Therefore, these explanations should be considered as hypotheses rather than conclusions derived from the data. The findings should be interpreted within the conceptual framework of personality-related belief patterns rather than formal personality traits or personality disorders, as assessed by the PBQ-SF. Accordingly, the observed associations should not be interpreted as evidence of underlying behavioral or mechanistic pathways.

The primary objective of diabetes management is to prevent acute and chronic complications while maintaining quality of life. Achieving this goal requires not only pharmacological optimization but also sustained behavioral engagement. Early identification of psychological and personality-related factors may therefore enhance treatment effectiveness [14]. In this context, the present findings contribute to the growing body of literature examining psychosocial determinants of glycemic regulation.

Notably, no significant differences were observed across glycemic control groups in terms of general psychopathological symptoms assessed by the SCL-90-R for both 1-year and 5-year HbA1c averages. This finding suggests that broad psychological distress, as measured by general symptom scales, may not directly correspond to glycemic status in T2DM. Previous studies have similarly indicated that while the SCL-90-R demonstrates construct validity across clinical populations, it may have limited sensitivity in detecting disease-specific psychological patterns related to chronic illness management [15,16]. Additional evidence suggests that general psychological symptom burden does not adequately explain variations in disease-related behaviors or metabolic outcomes [17]. Furthermore, although certain diabetes-related complications have been associated with increased psychological distress, this relationship appears complex and bidirectional rather than directly predictive of glycemic control [18,19]. Considering these findings, these findings suggest that general psychological symptom severity alone may not sufficiently account for differences in glycemic control. In contrast, personality-related belief patterns may offer additional, although still indirect, insight into behavioral processes relevant to disease management. However, these interpretations should be considered cautiously, as the present study did not include direct measures of self-management or adherence.

Our group comparisons revealed that specific personality-related belief patterns, particularly obsessive-compulsive, histrionic, paranoid, passive-aggressive, antisocial, and schizoid dimensions, differed significantly across HbA1c categories, with higher scores observed in the moderate control group. Previous research suggests that personality-related factors may influence disease perception, coping strategies, and engagement with diabetes care in T2DM [20,21]. In addition, maladaptive belief patterns have been associated with poorer self-care behaviors, while individual differences in impulsivity and cognitive styles may act as risk or resilience factors in diabetes self-management [22,23].

Positive psychological constructs such as self-efficacy and adaptive mindsets have been linked to improved glycemic outcomes, and psychological interventions may have beneficial effects on both metabolic and psychological parameters [24,25]. Furthermore, psychosocial stress, social support, and depression-related impairments in self-care behaviors have been identified as important factors influencing metabolic control [26,27]. Sustained engagement in diabetes self-management remains a key component of glycemic regulation [28].

However, these associations should be interpreted cautiously, as the present study did not include direct measures of self-management, adherence, or behavioral engagement. In addition, the literature remains heterogeneous, and some studies have reported no significant association between personality-related factors and glycemic control, possibly due to differences in study design, treatment characteristics, and population profiles [29].

An unexpected finding of this study was that several maladaptive personality-related belief scores were higher in patients with moderate glycemic control compared to those with poor control. This counterintuitive pattern may reflect the influence of unmeasured or residual confounding factors, such as differences in disease severity, treatment intensity, or psychosocial variables not captured in the present dataset. Alternative explanations should also be considered, including survivor effects, behavioral heterogeneity among patient groups, and differential engagement with healthcare services. In addition, measurement-related limitations of the PBQ-SF, which assesses belief patterns rather than stable personality constructs, may have contributed to this observation. Statistical instability related to subgroup sample sizes may also have influenced these results. Given these considerations, this finding should not be interpreted as a straightforward psychosocial pattern but rather as a complex and potentially unstable observation requiring further investigation.

Furthermore, given the large number of comparisons performed across multiple PBQ-SF and SCL-90-R subscales, the possibility of spurious statistically significant findings due to chance cannot be excluded. In the absence of formal correction for multiple testing, some observed associations may reflect type I error rather than true underlying relationships. Accordingly, these findings should be interpreted cautiously and considered exploratory, pending confirmation in larger studies with appropriate statistical adjustments.

Importantly, after adjustment for potential confounding factors including age, sex, diabetes duration, treatment regimen, and comorbidities, most associations were no longer statistically significant. This attenuation of effects following multivariable adjustment suggests that several of the associations observed in univariate or group-based analyses may not represent independent relationships. Only passive-aggressive personality-related belief patterns remained independently associated with HbA1c levels in the fully adjusted model. Accordingly, greater emphasis should be placed on this finding, while other observed associations should be interpreted with caution due to their lack of robustness after adjustment and the potential influence of multiplicity.

Passive-aggressive belief patterns are generally characterized by indirect resistance, procrastination, and difficulty complying with structured demands. However, any interpretation linking these patterns to behavioral mechanisms should be approached with caution, as the present study did not include direct measures of adherence or self-management behaviors. These features may hypothetically be relevant in the context of T2DM management, which requires sustained engagement with medication use, dietary regulation, glucose monitoring, and regular clinical follow-up.

The 5-year HbA1c analysis showed a similar pattern. Although general psychological symptoms did not differ across long-term glycemic categories, several maladaptive personality-related belief patterns were more pronounced in individuals with moderate glycemic control. Despite a higher proportion of patients meeting the predefined follow-up frequency threshold in this group, optimal metabolic targets were not achieved. This observation may indicate the influence of factors beyond healthcare utilization; however, such interpretations remain speculative given the available data.

Overall, these findings suggest that glycemic control in T2DM cannot be fully explained by pharmacological management or disease duration alone. Personality-related belief patterns may be associated with differences in glycemic outcomes; however, no direct clinical or behavioral implications can be inferred from the present findings.

### Limitations

This study has several limitations. Its retrospective cross-sectional, single-center design limits generalizability and precludes causal inference. The relatively small sample size in relation to multiple comparisons may reduce statistical power and increase the risk of type I error. Although key clinical variables were adjusted for, residual confounding and confounding by indication cannot be excluded. The use of self-report instruments (PBQ-SF and SCL-90-R) may introduce reporting bias, and HbA1c does not capture glycemic variability or hypoglycemia burden. In addition, HbA1c testing frequency was used as an indirect proxy for follow-up, which may reflect multiple factors beyond patient adherence. Several relevant confounders, including socioeconomic and behavioral variables, could not be assessed due to the retrospective design. Finally, the predominance of female participants and consecutive sampling may introduce selection bias.

## 5. Conclusions

This study identified associations between specific maladaptive personality-related belief patterns and glycemic control in patients with diabetes mellitus. Individuals with moderate glycemic control (HbA1c 7–9%) exhibited higher scores across several dimensions compared to those with poor glycemic control. However, most of these associations were not sustained after multivariable adjustment, with only passive-aggressive personality-related belief patterns remaining independently associated with HbA1c levels. No significant relationship was observed between general psychiatric symptom severity and glycemic outcomes. The observed pattern across glycemic control groups, including the higher scores in the moderate group, may reflect residual confounding or other unmeasured factors rather than a direct psychosocial mechanism. Overall, these findings suggest a potential association between personality-related belief patterns and glycemic control; however, they should be interpreted cautiously. The results are exploratory in nature, and no definitive conclusions can be drawn regarding behavioral mechanisms or clinical utility. Further longitudinal studies incorporating direct measures of self-management and adherence are needed to clarify these relationships.

## Figures and Tables

**Table 1:** Clinical and Psychological Characteristics According to 1-Year Mean HbA1c.

	HbA1c				
Parameters	Group 1 (<7) (n = 28)	Group 2 (7 ≤ HBA1c < 9) (n = 66)	Group 3 (≥9) (n = 80)	Total (n = 174)	*p*
**Age (Year) (Mean + SD)**	53.4 ± 11.5	54.2 ± 14.9	57.9 ± 14.1	55.8 ± 14.1	0.180
**Gender (%)**					0.983
**-Female**	20 (11.5%)	47 (27.0%)	56 (32.2%)	123 (70.7%)	
**-Male**	8 (4.6%)	19 (10.9%)	24 (13.8%)	51 (29.3%)	
**PERSONALITY BELIEF SCALE**					
**Avoidant Personality** **(Mean ± SD)**	12.7 ± 6.5	13.2 ± 6.4	11.8 ± 6.1	12.5 ± 6.3	0.425
**Dependent Personality** **(Median (IQR))**	9.0 (0.0–21.0)	9.0 (0.0–27.0)	10.6 (0.0–24.0)	9.7 (0.0–24.0)	0.750
**Passive–Aggressive Personality** **(Median (IQR))**	9.7 (0.0–24.0)	11.2 (0.0–28.0)	8.8 (0.0–20.0)	9.9 (0.0–28.0)	0.66
**Obsessive–Compulsive Personality (Mean ± SD)**	11.8 ± 4.9	13.9 ± 5.2	10.9 ± 4.3	12.2 ± 4.0	**0.041**
**Antisocial Personality** **(Median (IQR))**	7.5 (0.0–23.0)	10.2 (0.0–28.0)	9.5 (0.0–24.0)	9.7 (0.0–28.0)	0.453
**Narcissistic Personality** **(Mean ± SD)**	8.5 ± 2.9	9.9 ± 3.6	8.5 ± 3.3	9.0 ± 4.0	0.324
**Histrionic Personality** **(Median (IQR))**	4.3 (0.0–19.0)	7.5 (0.0–23.0)	5.8 (0.0–18.0)	6.2 (0.0–23.0)	**0.022**
**Borderline Personality** **(Median (IQR))**	10.0 (0.0–19.0)	10.5 (0.0–28.0)	10.5 (0.0–28.0)	10.0 (0.0–28.0)	0.551
**Schizoid Personality** **(Mean ± SD)**	12.8 ± 5.5	13.8 ± 5.6	12.3 ± 5.6	13.0 ± 6.0	0.307
**Paranoid Personality** **(Median (IQR))**	9.5 (0.0–26.0)	11.6 (0.0–28.0)	7.7 (0.0–26.0)	9.0 (0.0–26.0)	**0.047**
**Psychiatric Treatment (%)**					0.097
**-None**	21 (12.1%)	49 (28.2%)	70 (40.2%)	140 (80.5%)	
**-Yes**	7 (4.0%)	17 (9.8%)	10 (5.7%)	31 (19.5%)	
**Comorbid Medical Conditions (%)**					0.487
**-None**	7 (4.0%)	10(5.7%)	13 (7.5%)	30 (17.2%)	
**-Yes**	21 (12.1%)	56(32.2%)	67 (38.5%)	144 (82.8%)	
**HbA1c follow-up frequency threshold (%)**					**0.032**
**-Below threshold**	15 (8.6%)	44 (25.3%)	63 (36.2%)	122 (70.1%)	
**-Meets threshold**	13 (7.5%)	22 (12.6%)	17 (9.8%)	52 (29.9%)	
**Treatment Received (%)**					**<0.001**
**-OA**	21 (14.1%)	31 (17.8%)	13 (7.5%)	65 (37.4%)	
**-Insulin**	1 (0.6%)	8 (4.6%)	10 (5.7%)	19 (10.9%)	
**-Mix**	6 (3.4%)	27 (15.5%)	57 (32.8%)	90 (51.7%)	

Bold values indicate statistical significance (*p* < 0.05).

**Table 2:** Comparison of Clinical and Psychological Parameters According to 5-Year Mean HbA1c Levels.

	HbA1c				
Parameters	Group 1 (<7) (n = 26)	Group 2 (7 ≤ HbA1c < 9) (n = 65)	Group 3 (≥9) (n = 74)	Total (n = 165)	*p*
**Age (Year) (Mean + SD)**	53.0 ± 13.0	55.5 ± 13.6	58.0 ± 13.4	56.2 ± 13.4	0.229
**Gender (%)**					0.872
**-Female**	19 (11.5%)	45 (27.3%)	54 (32.7%)	118 (71.5%)	
**-Male**	7 (4.2%)	20 (12.1%)	20 (12.1%)	47 (28.5%)	
**PERSONALITY BELIEF SCALE**					
**Avoidant Personality** **(Mean ± SD)**	11.9 ± 4.6	14.2 ± 4.4	11.0 ± 4.0	12.4 ± 6.4	0.010
**Dependent Personality** **(Median (IQR))**	8.0 (0.0–21.0)	10.0 (0.0–27.0)	10.0 (0.0–24.0)	10.0 (0.0–27.0)	0.472
**Passive–Aggressive Personality (Median (IQR))**	9.0 (0.0–22.0)	12.0 (0.0–28.0)	9.0 (0.0–20.0)	10 (0.0–28.0)	**0.008**
**Obsessive–Compulsive Personality (Mean ± SD)**	12.1 ± 5.6	14.2 ± 4.6	10.6 ± 4.0	12.2 ± 4.1	**0.012**
**Antisocial Personality** **(Median (IQR))**	8.5 (0.0–23.0)	11.0 (0.0–28.0)	10 (0.0–20.0)	10.0 (0.0–28.0)	**0.034**
**Narcissistic Personality** **(Mean ± SD)**	8.1 ± 3.6	10.3 ± 3.4	8.4 ± 3.3	9.1 ± 4.0	0.112
**Histrionic Personality** **(Median (IQR))**	4.0 (0.0–21.0)	7.0 (0.0–23.0)	6.0 (0.0–18.0)	6.0 (0.0–23.0)	0.052
**Borderline Personality** **(Mean ± SD)**	8.9 ± 3.1	11.8 ± 3.4	10.5 ± 3.3.5	10.8 ± 3.8	0.097
**Schizoid Personality** **(Mean ± SD)**	12.4 ± 4.2	14.4 ± 4.0	11.9 ± 4.4	13.0 ± 4.4	**0.042**
**Paranoid Personality** **(Median (IQR))**	8.0 (0.0–26.0)	13.0 (0.0–28.0)	8.0 (0.0–26.0)	9.0 (0.0–28.0)	**0.003**
**Psychiatric Treatment (%)**					0.059
**-None**	19 (11.5%)	49 (29.7%)	66 (40.0%)	134 (81.2%)	
**-Yes**	7 (4.2%)	16 (9.7%)	8 (4.8%)	31 (18.8%)	
**Comorbid Medical Conditions (%)**					0.294
**-None**	6 (3.6%)	7 (4.2%)	13 (7.9%)	26 (15.8%)	
**-Yes**	20 (12.1%)	58 (35.2%)	61 (37.0%)	139 (84.2%)	
**HbA1c follow-up frequency threshold (%)**					**0.004**
**-Below threshold**	14 (8.5%)	42 (25.5%)	62 (37.6%)	118 (71.5%)	
**-Meets threshold**	12 (7.3%)	23 (13.9%)	12 (7.3%)	47 (28.5%)	
**Treatment Received (%)**					**<0.001**
**-OA**	18 (10.9%)	33 (20.0%)	12 (7.3%)	63 (38.2%)	
**-Insulin**	2 (1.2%)	7 (4.2%)	7 (4.2%)	16 (9.7%)	
**-Mix**	6 (3.6%)	25 (15.2%)	55 (33.3%)	86 (52.1%)	

Bold values indicate statistical significance (*p* < 0.05).

**Table 3:** Results of univariate and multivariable binary logistic regression analysis for the determination of personality traits associated with HbA1c levels.

Parameters	Univariate		Multivariable	
	*p* Values	OR (CI 95%)	*p* Values	OR (CI 95%)
**Passive-Aggressive Personality (per point increment)**	<0.001	1.12 (1.04–1.17)	0.008	1.12 (1.01–1.18)
**Obsessive-Compulsive Personality (per point increment)**	0.016	1.36 (1.15–3.27)	ns	
**Antisocial Personality (per point increment)**	0.012	1.74 (1.05–3.18)	ns	
**Schizoid Personality (per point increment)**	0.004	2.12 (1.18–3.72)	ns	
**Paranoid Personality (per point increment)**	<0.001	2.47 (1.64–3.87)	ns	

**Note:** Statistically significant results (*p* < 0.05). **Abbreviations:** ns, not significant; OR, Odds Ratio; CI, Confidence Interval.

## Data Availability

The datasets generated and/or analyzed during the current study are not publicly available due to privacy and ethical restrictions related to patient data but are available from the corresponding author on reasonable request.
